# Supplementary material for: Alcohol drinking delays the rate of sputum smear conversion among DR-TB patients in northwest Ethiopia; A retrospective follow-up study
Source: PLoS One. 2022 Mar 9;17(3):e0264852. doi: 10.1371/journal.pone.0264852 (PMC8906643; doi:10.1371/journal.pone.0264852)
Supplement: Attachment — Submitted filename: Comments for Author.docx [file pone.0264852.s002.docx]

**Comments for Author**

Title

- Title is not clear. Title of the study should reflect the outcome variable, population and study area. But the title of this topic lacks the description of outcome variable, population and study area. It needs modifications?
- I believe the main reason to put title in this way is due to the finding of your study. Only one factor is significantly associated with outcome variable. If so, what was new with your findings. Even the median time is within the expected time, what is the significance of this study for the existing knowledge/literature?
- What mean by retrospective follow up study?

Abstract

- The background section of abstract lacks a gap ( a gap in literature, gap in outcome measurement, a gap population, a gap in methodology….)
- Include the aim of the study/objective at the last paragraph Background

Methods

- Add “s” on method
- I think the method section lacks very important components of writing method in abstract, please re-write it (add study area, participants, study area, sample size and sampling techniques)

Results: add “s” on result

Conclusion

- … expected time frame…what was the expected time frame?
- Add recommendation based on the findings of your study. If your finding lack recommendation, your finding lacks signific

Introduction

- Introduction part is very shallow, it is not detail, please add by considering different literatures that focus on this area. What is the magnitude of the problem, where is the gap, what is the significance of conducting this study? If your introduction is not well stated, there is no way to convince the reader for the presence of the problem.
- The second paragraph of introduction cited with one reference, please revise the paragraph
- The third and fourth paragraph of introduction lacks references????
- Overall the introduction part need major revisions

Methods

- What is retrospective follow study?
- Line 89 and 90 of method part is not clear, please re-phrase it.
- Why u used September 2010 as baseline for study period?
- Second paragraph requires a references
- Operational definition need references
- Please explain method of model building

Result

- Briefly write how 436 patients cards included in the final analysis
- Adverse drug effect- what are the lists of ADE
- Scanty, grade 1+, grade 2+, and grade 3+ need operational definition
- What was the proportion censored outcome (for each censored categories)
- What was the incidence rate of smear conversion and person time observation?
- The final table need revision

Discussion

In second paragraph of discussion you stated that socio-demographic and other factors are the reason for discrepancy. What are the socio-demographic and other factors that contribute for the differences and how they could contributes for observed discrepancy?

How sample size increase or decrease the median time to sputum conversion. This is an awkward statement. Or clearly put justification to convince the reader.

Overall, the discussion section is very shallow and it needs major modification. It seems a report of some preliminary data, not the study conducted with advanced statistics.

Conclusion and recommendation

- In our study, the median time of sputum smear conversion was with in the expected time frame of conversion…what means by expected time frame of conversion. If it is with expected time frame what is new with this study?
- Please add the recommendation based your findings
